# Fluoroscopy-guided double-J ureteral stent removal using a novel metallic hook device “Kagibo” in women: A retrospective study

**DOI:** 10.1097/MD.0000000000042843

**Published:** 2025-06-13

**Authors:** Kanao Kobayashi, Kenichiro Ikeda, Norimasa Ogasawara

**Affiliations:** a Department of Urology, Chugoku Rosai Hospital, Japan Organization of Occupational Health and Safety, Hiroshima, Japan.

**Keywords:** DJ stent, Kagibo, women

## Abstract

The replacement of double-J ureteral stents (DJ stents) is a routine procedure in urology that conventionally requires cystoscopy. We developed a new medical device, the “Kagibo,” for removing DJ stents without cystoscopy in women. This study aimed to report the usefulness and safety of Kagibo in DJ stent removal in women. This retrospective study included a total of 34 female patients with a median age of 67 years (range: 44–87 years) who required DJ stent replacement between April and July 2024. The procedure was performed with the patients in the supine position without pain management. DJ stent replacement was performed as follows: the Kagibo was inserted into the bladder through the external urethral orifice; the distal end of the DJ stent was hooked to the Kagibo under fluoroscopic guidance; and the Kagibo was pulled outside the urethra, and the old DJ stent was replaced with a new stent. We defined success as cases in which DJ stents could be removed with the Kagibo and complications as cases requiring additional procedures or drug therapy associated with DJ stent removal. We retrospectively investigated the success rates and incidence of complications. The success rate of DJ stent removal using the Kagibo was 100%. No complications were observed. Our findings indicate that the Kagibo allows for the safe and effective removal of DJ stents without cystoscopy in women.

## 1. Introduction

A double-J (DJ) stent is a thin, flexible tube designed to maintain ureteral patency in various benign or malignant conditions. It is called a DJ stent because of its characteristic shape, with both ends curled in a J form. DJ stents require periodic replacement at least once every 6 months to maintain urinary patency, and cystoscopy is usually used for removal.^[1,2]^ DJ stent replacement is a routine procedure for urologists; however, the preparation involves setting up a cystoscope and various other items in a fluoroscopy suite, which can burden healthcare professionals.

Techniques have been reported to remove DJ stents under fluoroscopic guidance where a vascular sheath or Nelaton catheter is 1st inserted to introduce a snare loop or foreign body retrieval forceps.^[1–4]^ Special stents that require no cystoscopy or fluoroscopy have also been developed; however, magnetic stents^[5]^ are often expensive, and stents with extraction strings^[6–8]^ often result in dislodgement, neither of which has been widely used to date. Furthermore, Kawahara et al^[9]^ reported using a crochet hook to remove a DJ stent; however, the success rate was low. We also tried using a crochet hook and found it to be a useful tool for removing stents without using a cystoscope; however, certain aspects could be improved, such as the size and material, and fluoroscopy was expected to further improve the success rate. Moreover, because the crochet hook is not a medical device, continuing research using it is challenging. Therefore, we developed the “Kagibo” and registered it as a medical device.

This study aimed to determine whether the Kagibo is an effective and safe medical device for removing DJ stents without using a cystoscope in women.

## 2. Materials and methods

### 2.1. Participants

We included female patients who underwent DJ stent replacement between April and July 2024 and were scheduled for stent removal using the Kagibo. These patients were consecutively selected without exclusion. A total of 34 female patients were included in the study.

### 2.2. Technique

**Figure 1.**
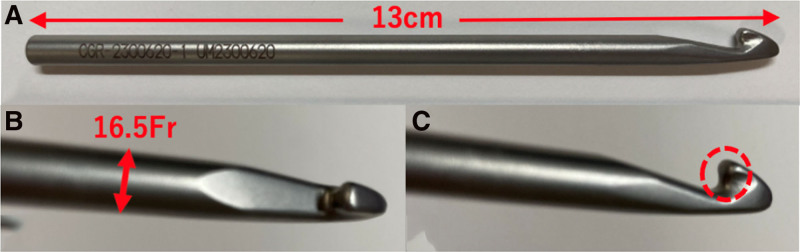
The Kagibo. The Kagibo is made of stainless steel, measuring 13 cm in length with a shaft size of 16.5 Fr. As shown within the red dotted circle, the hook is designed with a more obtuse angle compared to that of a crochet hook.

The Kagibo device used in this study was made of stainless steel, 13 cm in length, 16.5 Fr in shaft size, and weighed 24 g (Fig. 1A,B). The product was manufactured by Umihira Co., Ltd. (Kyoto, Japan), a company actively engaged in medical engineering collaborations, and was approved as a general medical device by the Pharmaceuticals and Medical Devices Agency in March 2024 (Notification No.: 26B2X10004002292). The Kagibo was sterilized with autoclaving prior to use.

Unlike a conventional crochet hook, the Kagibo is constructed from radiopaque stainless steel to ensure fluoroscopic visibility and features a larger shaft diameter (16.5 Fr vs 7.5 Fr) to improve handling. In addition, the hook angle was made more obtuse to reduce urethral discomfort during withdrawal and to enhance hooking efficacy for standard 6 Fr DJ stents.

**Figure 2.**
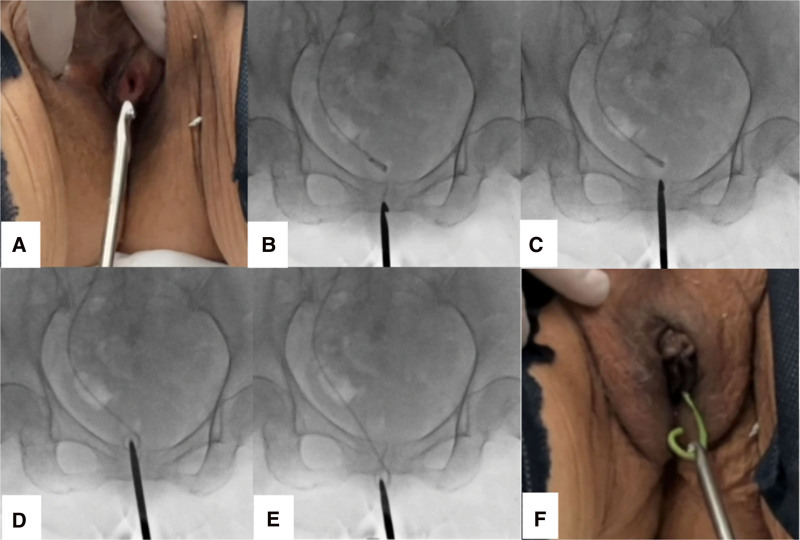
Steps of DJ stent removal using the Kagibo. The Kagibo is autoclave-sterilized before use. During the procedure, 2% lidocaine jelly is applied to the device tip before insertion into the external urethral orifice (A). Under real time fluoroscopic guidance, the Kagibo is advanced into the bladder (B, C) to hook the distal loop of the DJ stent (D), which is then withdrawn through the urethra for complete removal (E, F).

During the procedure, lidocaine 2% jelly was applied to the tip of the Kagibo before insertion into the external urethral orifice (Fig. 2A). While viewing real time fluoroscopic images, the Kagibo was advanced into the bladder (Fig. 2B,C) to hook the loop of the distal end of the DJ stent (Fig. 2D). It was then pulled outside through the external urethral orifice for complete removal (Fig. 2E,F). After stent removal, a new DJ stent was placed under fluoroscopic guidance using a standard technique. These techniques were performed with patients in the supine position without pain management, such as sedation, anesthesia, or suppositories. We defined success as cases where DJ stents could be removed with Kagibo and complications as cases requiring additional procedures or drug therapy following DJ stent removal. We retrospectively investigated the success rate and incidence of complications. Our Institutional Review Board reviewed and approved this study (Approval No. 2024-11). Informed consent was obtained through an opt-out process. A notice explaining the study’s purpose, methods, and data usage was posted on the hospital’s website. Patients could decline participation at any time by contacting the provided phone number. No patients opted out of this study.

This retrospective study did not include a control group using the conventional cystoscopic technique; however, comparative data from previous studies are discussed in the Discussion section.

## 3. Results

Characteristics of the patients and stents are summarized in Table 1. The median age was 67 years (range: 44–87). The affected side was the right ureter in 19 patients (55.9%) and the left in 15 patients (44.1%). The primary condition was urolithiasis in 31 cases (91.2%), followed by malignant tumors (5.9%) and ureteral stricture (2.9%). All patients received 6 Fr DJ stents, with the most commonly selected stent length being 24 cm (76.5%). The most frequently used stent type was Bard^®^ InLay Optima^®^ (C. R. Bard, Inc., Warwick) (73.5%), followed by Tria™ (Boston Scientific Corporation, Marlborough) (26.5%).

**Table 1:** Characteristics of the patients and stents.

Age, median (range)	67 (44–87)
Side, n (%)	
Right	19 (55.9%)
Left	15 (44.1%)
Primary disease, n (%)	
Urolithiasis	31 (91.2%)
Malignant tumor	2 (5.9%)
Ureteral stricture	1 (2.9%)
Stent diameter, n (%)	
6 Fr	34 (100%)
Stent length, n (%)	
24 cm	26 (76.5%)
26 cm	8 (23.5%)
Stent type, n (%)	
Bard^®^InLay Optima^®^	25 (73.5%)
Tria^TM^	9 (26.5%)

The success rate of DJ stent removal using the Kagibo was 100%. No cases of complications that required additional procedures or drug therapy were observed.

The median fluoroscopy time required for stent removal was approximately 20 seconds (range: 10–60 s), and the entire replacement procedure was typically completed within 5 minutes.

The cohort included 11 patients (32.3%) aged ≥75 years who were classified as the older group. The distribution of the affected sides was nearly equal (right: left = 19:15), and urolithiasis was the primary underlying condition in >90% of cases. All patients underwent the placement of a DJ stent per the institutional protocol. Regarding the DJ stent length, shorter stents (24 cm) were frequently selected (75%), likely due to the smaller stature of female patients. Regarding the DJ stent type, Bard^®^ InLay Optima^®^ was the most used (70%). Patients who experienced encrustation or obstruction with Bard^®^ InLay Optima^®^ had their stent switched to Tria™.

Additionally, the cohort included 6 patients (17.6%) on anticoagulant therapy, 8 patients (23.5%) with diabetes mellitus, 7 patients (20.6%) with a history of febrile urinary tract infections within the past year, and 6 patients (17.6%) with a stent indwelling period >3 months.

These findings indicated that the procedure was effective regardless of age, affected side, underlying disease, or DJ stent type. Furthermore, the procedure was confirmed to be safe even in patients with significant comorbidities, such as those on anticoagulant therapy, those with diabetes mellitus, and those with a history of urinary tract infections.

## 4. Discussion

Cystoscopy is the standard technique for removing DJ stents; however, the preparation requires setting up the cystoscope, arranging the irrigation fluid, a light source unit, grasping forceps, and other instruments in a fluoroscopy suite, which can place a substantial burden on healthcare staff, such as physicians or nurses.

In addition to techniques using grasping forceps or a snare catheter for fluoroscopy-guided DJ stent removal,^[1–4]^ an ultrasound-guided technique has been reported as a method for removing DJ stents without fluoroscopy or cystoscopy,^[10]^ yielding favorable outcomes regarding the success rate and incidence of complications. However, the handmade spiral-ending device used in the study did not gain widespread use. Another method using a special magnetic DJ stent was reported to be less painful and faster than standard cystoscopic removal.^[5]^ However, magnetic DJ stents are not commonly used because they cost 10,000 to 30,000 JPY more than standard DJ stents and are contraindicated for magnetic resonance imaging. Furthermore, ureteral stents with extraction strings have been reported; despite them being less painful and highly cost-saving,^[7,8]^ they have a high rate of dislodgement among women (approximately 25%),^[6]^ limiting their practical use.

Kawahara et al reported a crochet hook technique, which is limited to women, as a method of DJ stent removal that does not require fluoroscopy or cystoscopy.^[9]^ However, the crochet hook is intended for knitting to create fabric or patterns from yarn; in light of the Clinical Trials Act, it could not continue to be used in real-world clinical settings. Therefore, we decided to develop the Kagibo as a medical device based on a crochet hook. As the success rate was limited (83.9%) with the crochet hook technique,^[9]^ we made several improvements to the Kagibo. As DJ stent placement is performed under fluoroscopic guidance, the minimal use of fluoroscopy for the preceding DJ stent removal was considered acceptable. Hence, radiopaque stainless steel was adopted to ensure good visibility, even under spot fluoroscopy, while minimizing radiation exposure. In addition, the crochet hook had a small diameter (7.5 Fr),^[9]^ whereas the diameter of the Kagibo was increased to match that of a flexible cystoscope (16.5 Fr) to enhance the ease of hooking a standard 6 Fr stent. Furthermore, to reduce the tugging sensation or discomfort in the urethra caused by the stent withdrawal, the angle of the hook was made more obtuse than that of the crochet hook, as indicated by the red dotted circle in Figure 1C.

An advantage of using this technique is that the stent is grasped by hooking its distal end to avoid blindly damaging the bladder mucosa, as with foreign body forceps.^[1,3]^ Unlike snare forceps, there is also no need to fill the bladder with urine or saline solution containing a contrast agent to keep the stent visible.^[1–4]^ Additionally, when the tip of the stent is located at the base of the urinary bladder, the snare forceps need to be pushed to the upper wall of the bladder so that it curves downwards.^[1,3]^ With our technique, the stent can be easily captured by advancing the Kagibo towards the distal end of the stent under fluoroscopic guidance and adjusting the angle upwards or downwards. According to 3 urologists at our hospital who have used this technique, several experiences are sufficient to master the technique. Considering that they were each in their 26th, 19th, and 2nd years of professional experience, the difficulty level of the technique was not considered high.

In this study, stent removal using the Kagibo was successful in all cases. However, Kawahara et al reported that the success rate with the Kagibo was low among cases with a poor stent position.^[9]^ For that reason, when the distal end of a stent is not in a pigtail shape but short and straight, removing the stent may be difficult with our technique. In addition, the removal of a migrated stent or a stent with the entire distal end embedded due to calcification is expected to be a challenge; however, this is also likely to be true for the cystoscopic technique.

Recent studies have investigated alternative techniques that reduce reliance on cystoscopy. Lim et al introduced an “alternative snare technique,” employing a 0.018-inch guidewire and a 5-Fr catheter to create a snare for DJ stent removal.^[11]^ This technique demonstrated a technical success rate of 72.7% in their cohort but faced limitations in cases with stent migration or significant obstruction by debris. Similarly, Chen et al reported a non-cystoscopic method for removing DJ stents using a simple device consisting of a feeding tube and suture material.^[12]^ This method achieved a 97.8% success rate while significantly reducing the procedure time and hospitalization costs compared with cystoscopy. However, multiple attempts are sometimes required when the distal stent tip is not adequately exposed. Using fluoroscopy to identify the location of the distal end of the stent before starting the procedure could help predict the need for cystoscopy.

Our technique did not result in any complications requiring additional treatment or procedures. Gross hematuria was observed in approximately half of the patients after stent replacement; however, this also occurred after cystoscopic stent replacement. Kawahara et al reported a case of urosepsis following stent removal with a crochet hook; however, they inferred that it was highly likely secondary to percutaneous nephrolithotomy with infected renal stones.^[9]^

While the Kagibo has the same shaft size as a 16.5 Fr flexible cystoscope, its tip is rounder than that of a cystoscope. Therefore, the tolerability of insertion may be higher. Kawahara et al reported that the visual analog pain scale score for stent removal using a crochet hook was significantly lower than that observed with stent removal using cystoscopy.^[9]^ In addition, by not using cystoscopy, the cost can be reduced by approximately 100 EUR according to Rassweiler et al^[5]^ and by approximately 100 USD according to Chang et al^[3]^ As of 2024, Japan’s public health insurance system requires the so-called working generation (<70 years of age) to pay 30% of their total medical bills. Based on this, the cost of ureteral stent replacement was calculated as 10,200 JPY (approximately 66 USD). By a simple calculation, subtracting the cost of flexible cystoscopy by 2850 JPY (approximately 19 USD) leads to a cost reduction of approximately 30%. Furthermore, a major advantage of not using cystoscopy is that it saves healthcare staff from the difficulty of preparing various items and instruments, including cystoscopes. It would be desirable for the Kagibo to eventually become a common medical device, similar to metal bougies, and always be kept in the operating room and outpatient clinics.

The main limitations of this study are that it was a retrospective study involving a small sample size, and no comparison was performed with the conventional cystoscopic technique. However, the Kagibo has many advantages, such as not requiring cystoscopy, technical simplicity, reliability, and high cost-effectiveness (reduced cost and burden of preparation). However, further studies are required to verify the efficacy and safety of this technique.

In conclusion, fluoroscopy-guided ureteral stent removal using the Kagibo is an effective, easy, and safe technique for women. The advantage of not requiring cystoscopy is considered substantial for both patients and healthcare professionals. To further enhance the applicability and utility of this technique, future studies should focus on comparing its outcomes with those of conventional cystoscopic methods in larger multi-institutional cohorts. Additionally, investigating its feasibility and safety in patients with complex conditions, such as severely encrusted or migrated stents, could broaden its indications. Exploring the cost-effectiveness of different healthcare systems may also provide valuable insights for its wider implementation. These studies would not only validate the findings of this study but also help refine and optimize the use of the Kagibo in clinical practice.

## Acknowledgments

We would like to thank Honyaku Center Inc. for English language editing.

## Author contributions

**Conceptualization:** Kanao Kobayashi.

**Data curation:** Kenichiro Ikeda.

**Formal analysis:** Kanao Kobayashi, Kenichiro Ikeda, Norimasa Ogasawara.

**Writing – original draft:** Kanao Kobayashi, Kenichiro Ikeda, Norimasa Ogasawara.

**Writing – review & editing:** Kanao Kobayashi, Norimasa Ogasawara.
